# Differences in levels of inflammatory mediators in meniscal and synovial tissue of patients with meniscal lesions

**DOI:** 10.1186/s40634-016-0041-9

**Published:** 2016-02-03

**Authors:** Takahiro Ogura, Miyako Suzuki, Yoshihiro Sakuma, Kazuyo Yamauchi, Sumihisa Orita, Masayuki Miyagi, Tetsuhiro Ishikawa, Hiroto Kamoda, Yasuhiro Oikawa, Izumi Kanisawa, Kenji Takahashi, Hiroki Sakai, Tomonori Nagamine, Hideaki Fukuda, Kazuhisa Takahashi, Seiji Ohtori, Akihiro Tsuchiya

**Affiliations:** Funabashi Orthopaedic Hospital Sports Medicine Center, Funabashi, Chiba Japan; Department of Orthopaedic Surgery, Graduate School of Medicine, Chiba University, Chiba, Japan

**Keywords:** Meniscal injury, Inflammatory mediator, Synovium, Osteoarthritis

## Abstract

**Background:**

Meniscal injuries are a risk factor for osteoarthritis (OA). While a mechanical pathway between meniscal injury and OA has been described, the biological effects of inflammation on this pathway have yet to be clarified. The aim of our study was to compare levels of specific inflammatory mediators, tumor necrosis factor-alpha (TNF-α), interleukin-6 (IL-6), and nerve growth factor (NGF), in injured and uninjured meniscal tissue and related knee joint synovium.

**Methods:**

Tissue samples were obtained from 19 patients, 31.1 ± 13.6 years old, who underwent arthroscopic partial meniscectomy. For analysis, tissue samples were categorized into the following groups: injured meniscal site (IM), non-injured meniscal site (NIM), synovium ‘nearest’ the lesion (NS), and synovium from the opposite knee compartment, ‘farthest’ synovium (FS). Levels of inflammatory mediators were determined using enzyme-linked immunosorbent assay and between-group differences (IM and NIM; NS and FS) were evaluated using the Wilcoxon signed-rank test. The association between pre-operative pain score and the level of each inflammatory mediator was evaluated using Spearman’s correlation.

**Results:**

Higher levels of TNF-α and IL-6 were identified in the IM tissue, compared to NIM (*p* <0.05). IL-6 levels were also higher in the NS compared to the FS (*p* <0.05). There was no correlation between pre-operative pain score and level of each inflammatory mediator.

**Conclusions:**

Our outcomes confirm a local increase in inflammatory mediator levels, in both meniscal and synovial tissue, which could contribute to development of OA. Management of these biological effects of meniscal injury might be warranted.

## Background

The menisci of the knee contribute significantly to the biomechanics of the knee joint, being important for load transmission, optimizing bone-on-bone contact and gliding movements, as well as working with the ligaments of the knee, including the anterior cruciate ligament, to provide joint stability (Kennedy et al. 1982; Seedhom et al. 1974). In view of the structural and functional importance of the menisci, surgical management of meniscal injuries, which have become very common among professional and amateur athletes, is recommended and are in fact among the most common indications for knee surgery (Sihvonen et al. 2013).

Different studies have reported meniscal injury to be a risk factor for the development of knee joint osteoarthritis (OA) (Englund et al. 2009; Ding et al. 2007; Zeichen et al. 2006). The association between meniscal injury or meniscectomy and knee OA has largely been investigated from a biomechanics perspective, that is, how altered stability and joint loading contribute to the disease process of OA. However, the biological effects of meniscal injury on the tissues of the knee joint remain unclear. In order to develop optimal therapeutic strategies, both the mechanical and biological pathways linking meniscal injury to knee OA must be understood.

The role of several inflammatory mediators in the disease process of knee OA has been studied, including tumor necrosis factor-alpha (TNF-α), interleukin-6 (IL-6) and nerve growth factors (NGF) (Fiorito et al. 2005; Pearle et al. 2007; Smith et al. 1997). For example, high concentration of TNF-α and IL-6 in synovial fluid were associated with early OA in an animal study (Venn et al. 1993). TNF-α can induce synovial cells and chondrocytes to produce IL-6, IL-8 and leukocyte inhibitory factor, as well as stimulate protease and prostaglandin production (Pelletier et al. 2001; Fernandes et al. 2002). High serum level of IL-6 has been reported to be a significant predictor of radiographic evidence of knee OA (Livshits et al. 2009). In addition, NGF was reported to be upregulated in chondrocytes and synovial fibroblasts in human OA joints, suggesting its crucial role in the pathology of OA (Iannone et al. 2002; Manni et al. 2003). Despite the important effect of these mediators on OA, the specific effect of inflammatory mediators associated to meniscal tears on meniscal and synovial tissue has yet to be clearly determined. In this study, we tested the hypothesis that injury to the meniscus would produce a local inflammatory response, involving both the meniscus and adjacent synovium, which could contribute to the development of OA. This hypothesis was evaluated by comparing levels of specific inflammatory mediators, TNF-α, IL-6, and NGF, in injured and uninjured meniscal tissue and related knee joint synovium.

## Methods

### Selection of the study group

Prospective participants were patients who were evaluated at our hospital for a history of ‘knee pain’, between November 2011 and May 2012, and who were diagnosed as having sustained a lesion in the avascular zone of the medial or lateral meniscus of the knee. The diagnosis was formed from the medical history, physical examination and magnetic resonance imaging (MRI). Prospective participants were further assessed to exclude concomitant ligament deficiency, knee joint OA and injury to the articular cartilage. OA and articular cartilage injury were excluded on MRI preoperatively and by arthroscopy intraoperatively. Nineteen patients met our inclusion and exclusion criteria, provided informed consent, and were enrolled in the study. These patients subsequently underwent arthroscopic partial meniscectomy. The methods and procedures were approved by our institutional review board.

### Pain evaluation

Prior to arthroscopy, patients were asked to rate the level of their knee pain, both at rest and during movement, on a 10-point numerical rating scale (NRS), with a score of ‘0’ indicating ‘no pain’ and a score of ‘10’ indicating the ‘worst pain imaginable’.

### Arthroscopic procedure

**Fig. 1:**
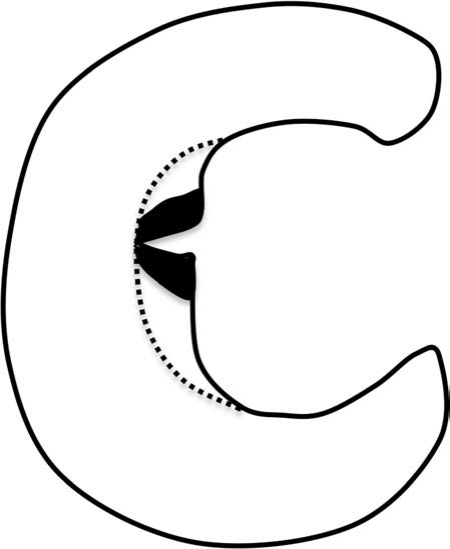
Schematic view of the injured meniscus (IM) and the non-injured meniscus (NIM). *Dotted line* indicates the resection line of meniscal tissue. IM includes tissue from the site of the tear (*black lesion*); NIM does not contain any tissue from the site of the tear (*white lesion* within the resected meniscal tissue)

The injured meniscal tissue was resected using an oval punch, as per routine procedures for arthroscopic partial meniscectomy. Additional meniscal tissue around the lesion was resected to obtain a smooth meniscal surface. During the arthroscopic procedure, we differentiated *injured* and *uninjured* meniscal tissue. Meniscal tissue from two separate areas were resected and classified into two groups for analysis: injured tissue from the meniscal site (IM), which included the meniscal lesions, and meniscal tissue from the non-injured site (NIM), which did not include tissue from the site of the meniscal lesion (Fig. 1). Both IM and NIM were obtained from only the avascular zone of the meniscus. Samples of the synovium were obtained from two sites for analysis: the ‘nearest’ synovium (NS) was attached to the joint capsule near the site of the meniscal injury, and the ‘farthest synovium’ (FS) was obtained from the opposite joint compartment (i.e., if the injury was to the medial meniscus, the synovium was obtained from the lateral compartment of the knee). Each sample included at least two or three pieces of tissue obtained using an oval punch.

Meniscal and synovial tissue samples were immediately stored at −80 °C. In preparation for analysis, the samples were thawed and subsequently diced and lysed with 500 μL of CelLytic M Cell Lysis Reagent. Inflammatory mediator quantification was performed using a double-antibody sandwich enzyme-linked immunosorbent assays (ELISA) for TNF-α, IL-6 (R&D systems, Minneapolis, MN) and NGF (Boster Biological Tec, Wuhan, China), using the manufacturers’ protocols. Tissue protein was assayed using the Bio-Rad kit (Bio-Rad Laboratories, Hercules, CA), again using the manufacturer’s protocols. Levels of inflammatory mediators were normalized to the protein level of each tissue (Jonas et al. 2015; Miyagi et al. 2011).

### Statistical analysis

Descriptive statistics were calculated for each measured variable. Differences in concentration of inflammatory mediators (TNF-α, IL-6 and NGF) between IM and NIM tissue samples, as well as between NS and FS, were evaluated using the Wilcoxon signed-rank test. A subanalysis was performed by sex and delay from the time of injury to surgery: acute group, less than 1 month from injury to surgery, and chronic group, more than 1 month from injury to surgery. The correlation between NRS score and the level of each inflammatory cytokine was evaluated using Spearman’s rank correlation. The level of significance was set at *p* <0.05, a priori. All statistical analyses were performed with Stata (version 13; StataCorp LP, College Station, TX).

## Results

### Description of the study group

The study group consisted of 12 men and 7 women, with a mean age of 31.1 ± 13.6 years. The mean time to surgery after injury was 7.7 months, with a range of 2 weeks to 3 years. In this group, 5 patients underwent surgery less than 1 month after injury, forming the acute group (mean delay, 3.2 weeks; range, 2 weeks to 1 month), and the other 14 patients more than 1 month after injury, forming the chronic group (mean delay, 10.1 months; range, 2 month to 3 years). With regards to the NRS score, 7 patients reported pain at rest, with a mean NRS score of 1.8 ± 2.3, and all patients reported pain on movement, with a mean NRS score of 6.5 ± 2.0 (Table 1).

### Concentrations of inflammatory mediators in the meniscus

**Fig. 2:**
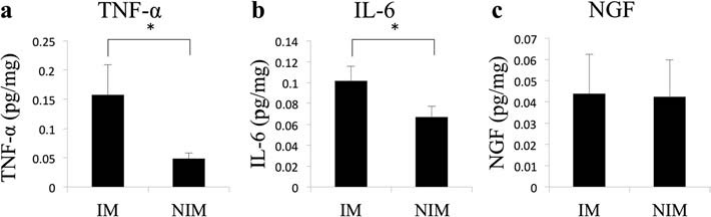
Levels of inflammatory mediators in meniscal tissues **a** tumor necrosis factor-alpha (TNF-α) and **b** interleukin-6 (IL-6), showing the significant elevation of levels in injured meniscal tissue (IM), compared to levels in non-injured tissue (NIM); **c** Comparable levels of nerve growth factor (NGF) in both IM and NIM tissues is shown; values are reported as a mean ± SD; * *p* <0.05

Compared to the NIM tissue, TNF-α levels were elevated in the IM tissue IM,0.16 ± 0.052 pg/mg; NIM, 0.048 ± 0.0096 pg/mg; *p* = 0.028; Fig. 2a). IL-6 levels were also elevated in the IM tissue, compared to NIM tissue (IM, 0.11 ± 0.014 pg/mg; NIM, 0.067 ± 0.0099 pg/mg; *p* = 0.038; Fig. 2b). Levels of NGF were comparable between the two meniscal tissues (IM, 0.044 ± 0.0042; NIM, 0.042 ± 0.0040 pg/mg; *p* = 0.31; Fig. 2c).

### Concentrations of inflammatory mediators in the synovium

**Fig. 3:**
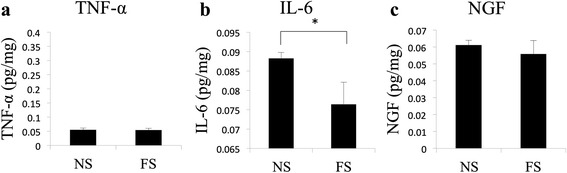
Concentrations of inflammatory mediators in the synovium, **a** showing the significant elevation of interleukin-6 level in the nearest synovial tissue (NS); levels of **b** tumor necrosis factor-alpha (TNF-α) and **c** nerve growth factor (NGF) are comparable in IM and NIM tissues; values are reported as a mean ± SD; * *p* <0.05

IL-6 levels were elevated in the NS synovial tissue, compared to the FS (NS, 0.088 ± 0.0015 pg/mg; FS, 0.076 ± 0.0057 pg/mg; *p* = 0.039; Fig. 3b). Levels of TNF-α and NGF were comparable in both NS and FS tissues (Fig. 3a, c).

### Correlation between NRS and levels of inflammatory mediators

The correlation between NRS and level of each inflammatory mediator is reported in Table 2. No correlation was identified between these parameters.

**Table 1:** Relevant demographic features of our study group

Case	Age	Sex	BMI	Side of meniscus	Mean time to surgery (months)	Tear pattern	NRS
							At rest/during movement
1	15	f	20.3	Lateral	2	Radial	0/8
2	40	m	26.6	Medial	12	Horizontal	6/5
3	37	m	26.8	Medial	4	Radial	0/5
4	18	m	20.2	Lateral	1	Radial	0/5
5	16	f	19.3	Lateral	3	Radial	6/7
6	44	f	21.9	Lateral	36	Radial	0/5
7	18	m	20.8	Lateral	1	Radial	0/7
8	41	m	24.1	Medial	0.5	Horizontal	0/5
9	39	m	22.8	Lateral	2	Radial	0/4
10	32	m	21.0	Medial	15	Horizontal	8/8
11	37	m	19.5	Lateral	4	Radial	0/10
12	23	f	21.7	Lateral	7	Radial	4/5
13	20	m	24.1	Lateral	2	Radial	0/7
14	35	m	21.5	Lateral	24	Radial	0/3
15	33	f	28.9	Medial	1	Horizontal	3/6
16	59	m	25.8	Medial	5	Radial	2/8
17	54	f	25.2	Medial	24	Radial	0/8
18	17	m	25.9	Lateral	2	Radial	0/8
19	13	f	18.4	Lateral	0.5	Radial	6/10

*BMI* body mass index, *f* female, *m* male, *NRS* numerical rating scale

**Table 2:** Correlation between Pre-operative NRS and Levels of Inflammatory Mediators

		TNF-α		IL-6		NGF	
		γ	*p* - value	γ	*p* - value	γ	*p* - value
NRS	Meniscus	0.3	0.21	0.36	0.13	−0.19	0.25
	Synovium	0.046	0.86	−0.029	0.91	−0.31	0.74

*NRS* numerical rating scale, *TNF-α* tumor necrosis factor-alpha, *IL-6* interleukin-6, *NGF* nerve growth factor

### Subanalysis

Results of our subanalysis are reported in Tables 3 and 4. No significant between-group or between-sex differences were identified.

**Table 3:** Level of inflammatory mediators in acute and chronic group

		Meniscus (pg/mg)			Synovium (pg/mg)		
		IM	NIM	*p* - value	NS	FS	*p* - value
		Mean	Mean				
TNF-α	Acute	0.44 ± .14	0.044 ± .007	.043	0.055 ± .004	0.053 ± 0.053	n.s.
	Chronic	0.059 ± .042	0.049 ± .022	.03	0.055 ± .006	0.055 ± .006	n.s.
IL-6	Acute	0.12 ± .03	0.055 ± .014	.043	0.087 ± .02	0.051 ± .14	.043
	Chronic	0.096 ± .017	0.071 ± .012	.03	0.088 ± .007	0.082 ± .014	.015
NGF	Acute	0.049 ± .082	0.042 ± .084	n.s.	0.053 ± .01	0.061 ± .017	n.s.
	Chronic	0.042 ± .004	0.042 ± .005	n.s.	0.057 ± .02	0.061 ± .03	n.s.

*TNF-α* tumor necrosis factor-alpha, *IL-6* interleukin-6, *NGF* nerve growth factor, *IM* injured meniscal site, *NIM* non-injured meniscal site, *NS* synovium ‘nearest’ the lesion, *FS* ‘farthest’ synovium, *n.s*. not significantly different, *SD* standard deviation

**Table 4:** Level of inflammatory mediators in male and female

		Meniscus (pg/mg)			Synovium (pg/mg)		
		IM	NIM	*p* - value	NS	FS	*p* - value
		Mean ± SD	Mean ± SD		Mean ± SD	Mean ± SD	
TNF-α	Male	0.12 ± .053	0.048 ± .03	.018	0.055 ± .002	0.055 ± .002	n.s.
	Female	0.23 ± .11	0.046 ± .004	.04	0.054 ± .002	0.053 ± .002	n.s.
IL-6	Male	0.079 ± .069	0.068 ± .014	.049	0.088 ± .0021	0.075 ± .0072	.003
	Female	0.14 ± .034	0.065 ± .012	.018	0.089 ± .0034	0.072 ± .011	.018
NGF	Male	0.041 ± .005	0.041 ± .006	n.s.	0.061 ± .001	0.054 ± .003	n.s.
	Female	0.044 ± .0072	0.048 ± .0067	n.s.	0.062 ± .012	0.06 ± .02	n.s.

*TNF-α* tumor necrosis factor-alpha, *IL-6* interleukin-6, *NGF* nerve growth factor, *IM* injured meniscal site, *NIM* non-injured meniscal site, *NS* synovium ‘nearest’ the lesion, *FS* ‘farthest’ synovium, *n.s*. not significantly different, *SD* standard deviation

## Discussion

The development of knee OA after meniscal injury is a significant health problem, leading to pain and limitations in activities and participation. Although the contribution of effects of altered knee mechanics, resulting from meniscal injury and meniscectomy, on the development of OA have been investigated, the effects of associated biological factors on the disease process of OA following injury have not been addressed. To this end, we investigated the difference in inflammatory mediator levels, perioperatively, in injured and non-injured meniscal tissue, as well as in the associated synovial tissue. Our study demonstrated a significant elevation of TNF-α and IL-6 cytokines in the injured meniscal tissue, with a concomitant increase of IL-6 in the synovial tissue local to the site of injury. This is an important finding as inflammatory mediators have been reported to play an important role in the development of knee OA (Edd et al. 2015; Olson et al. 2014). TNF-α directly stimulates sensory neurons, via its receptors, which initiates a cascade of inflammatory responses mediated through the production of ILs, including IL-1 and IL-6 (Ohtori et al. 2004; Pollock et al. 2002). In particular, there is evidence for a complex role of IL-6 in the pathogenesis of OA, mediated specifically through its effect on the production of tissue inhibitors of metalloproteinases-1 (MMP-1), which might influence the extent of cartilage damage, post-injury, through negative feedback (Lotz and Guerne. 1991). Thus, our identification of an elevation in TNF-α and IL-6 at the site of injury is indicative of a local inflammatory response which could initiate the cycle of cartilage damage leading to the development of early knee OA in the area local to the injury. As our intent was to clarify differences in inflammatory mediators induced by the injury, we obtained and evaluated tissue samples from the avascular zone of the meniscus. Although our findings cannot be extrapolated to tissue from the vascular zone of the meniscus, our use of avascular tissue prevented possible blood contamination, which would have made accurate comparison difficult.

Previous studies have reported elevations of TNF-α and IL-6 in the joint fluid after acute knee injury, including injury to the meniscus and to the anterior cruciate ligament (Tang et al. 2009; Higuchi et al. 2006; Irie et al. 2003). Although we evaluated inflammatory responses specifically in meniscal and synovial tissue, findings from these previous studies are consistent with ours. It is possible that inflammatory mediators produced by injured meniscal tissue could be one of the origins contributing to a general inflammatory response of the knee joint after meniscal injuries. Age may be an important factor influencing the inflammatory response of tissues following meniscal injury. Specifically, Brophy et al. have recently reported an elevation in the gene expression of arthritis-related markers, including a disintegrin and metalloproteinase with thrombospondin motifs 5 (ADAMTS-5), MMP-1 and MMP-13 in patients with meniscal injuries under the age of 40 years (Brophy et al. 2012). These findings provide evidence of clinically relevant, age-dependent differences in the response of the knee to meniscal tears, with elevated expression of arthritis-related markers, indicative of an increased catabolic response, in patients under 40 years of age. Future research is required to evaluate differences in gene expression of arthritis markers in injured and non-injured meniscal tissue to fully characterize the role of genetic factors in the disease pathway relating meniscal injuries to early onset of knee OA. Our findings demonstrating significant elevation of inflammatory mediators in injured and uninjured meniscal tissue, and in the synovium local to the injury, provide justification for future studies to clarify the role of this local inflammatory response of damaged meniscal tissue on the development of OA.

We did not identify a correlation between preoperative NRS scores and levels of the different inflammatory mediators evaluated. A relationship between the level of inflammatory mediators and patient-reported pain scores have, however, been reported by other researchers. Cuellar et al. reported a correlation between levels of IL-6 in synovial fluid and pain score in patients with meniscal tear, whereas there was no evidence of an effect of levels of TNF-α (Cuellar et al. 2009). Similarly, Cuellar et al. reported a correlation between levels of IL-6 in synovial fluid and pain score in patients with tears of the anterior cruciate ligament, with no effect associated to TNF-α levels (Cuellar et al. 2010). In contrast, Orita et al. reported a positive correlation between level of TNF-α in synovial fluid and pain score in patients with OA, with no effect of levels of IL-6 (Orita et al. 2011). Interestingly, among patients undergoing total knee replacement, a two-fold increase from baseline in plasma IL-6 levels was reported, post-surgery, which had no correlation to knee pain scores (Feng et al. 2008). Therefore, it is possible that the correlation between levels of inflammatory mediators and patient reported pain may be disease-dependent, which might explain the absence of a positive correlation in our study. The absence of a correlation could also result from the absence of sensory nerve endings in our sample meniscal tissues, which were obtained from the avascular zone of the meniscus. Previous study found that nerve fibers and sensory receptors mainly exist in the periphery and vascular zone of the meniscus, representing the outer one-third of the meniscus (Day et al. 1985). In addition, we did not identify an elevation of NGF level in the injured meniscal tissue, an inflammatory cytokine that specifically stimulates sensory nerve growth. The absence of an influence of meniscal injury on NGF could further explain the absence of sensory nerve growth in the injured tissue. Future studies on the association between meniscal injury, inflammatory response, and pain should consider the role of other molecules, such as prostaglandins, cyclooxygenase, and nitric oxide.

We also did not identify a correlation between elevated IL-6 in the synovium membrane and knee pain scores. This finding is corroborated by that of Scanzello et al.’s study indicating no correlation between the absence or presence of synovitis and preoperative pain scores (Scanzello et al. 2011). However, Scanzello et al. did report a positive correlation between presence of joint synovitis and functional score on the Lysholm scale. Therefore, it is likely that inflammation of the synovium affects knee function to a greater extent than pain. Future studies investigating the role of inflammation on knee pain and function should include scales of knee function, such as the Knee injury and Osteoarthritis Outcome Score (KOOS) and Lysholm scales.

Taken together, our results potentially indicate that a meniscal injury results in a significant elevation in the expression of the inflammatory mediators TNF-alpha and IL-6 locally at the site of injury, and of IL-6, in the synovium in close proximity to the injury. These local inflammatory responses could initiate the process of articular cartilage degeneration. However, the limitations of our study need to be noted in the interpretation of our results. Foremost, although we separated meniscal tissue from injured and uninjured sites for analysis, it is important to recognize that all sampled were obtained from patients with meniscal injury. No published data were identified in healthy menisci for comparison. Also, a variety of other factors could influence the level of inflammatory mediators in meniscal tissue, such as general health conditions, body mass index, smoking status, and age. Finally, extracellular signaling molecules are sometimes found attached to the extracellular matrix in injured joints. Therefore, it is possible that the cellular origins of inflammatory mediators may not be detectable by ELISA. In order to confirm whether these molecules are produced by the meniscus and synovium, quantitative real-time reverse transcription polymerase chain reaction (RT-PCR) analysis will be needed. However, we note the presence of RNA may not always accurately reflect tissue protein levels, which is a major disadvantage of RT-PCR analysis and why we used ELISA in our study, as it allowed us to identify secreted cytokines at the protein level.

## Conclusions

Our findings provide Level III evidence of a specific role of local inflammatory mediators in the disease process of meniscal lesions, which might contribute to the development of knee OA. Specifically, levels of inflammatory mediators TNF-α and IL-6 increased significantly at the site of the meniscal lesion, and IL-6 levels in the local synovium. Based on these results, we propose that management of these biological effects of meniscal injury might be warranted. However, future studies are required to evaluate the possible interaction between these biological effects on the mechanical pathway for the development of knee OA after meniscal injuries.

## Abbreviations

OA: osteoarthritis
TNF-α: tumor necrosis factor-alpha
IL-6: interleukin-6
NGF: nerve growth factor
IM: injured meniscal site
NIM: non-injured meniscal site
NS: synovium ‘nearest’ the lesion
FS: ‘farthest’ synovium
NRS: numerical rating scale
ELISA: enzyme-linked immunosorbent assays
ADAMTS-5: a disintegrin and metalloproteinase with thrombospondin motifs 5
MMP: matrix metalloproteinase
RT-PCR: reverse transcription polymerase chain reaction
